# Mesenchymal stem cell-derived exosomes for treatment of sepsis

**DOI:** 10.3389/fimmu.2023.1136964

**Published:** 2023-04-26

**Authors:** Kento Homma, Nikolay Bazhanov, Kazuki Hashimoto, Masaru Shimizu, Thomas Heathman, Qi Hao, Ranjana Nawgiri, Vidarshi Muthukumarana, Jae Woo Lee, Donald S. Prough, Perenlei Enkhbaatar

**Affiliations:** ^1^ Department of Anesthesiology, University of Texas Medical Branch, Galveston, TX, United States; ^2^ Department of Anesthesiology, University of California, San Francisco, CA, United States; ^3^ Department of Pathology, University of Texas Medical Branch, Galveston, TX, United States

**Keywords:** mesenchymal stem cell-derived exosomes, mesenchymal stem cell, exosome, sepsis, ovine model

## Abstract

**Introduction:**

The pathogenesis of sepsis is an imbalance between pro-inflammatory and anti-inflammatory responses. At the onset of sepsis, the lungs are severely affected, and the injury progresses to acute respiratory distress syndrome (ARDS), with a mortality rate of up to 40%. Currently, there is no effective treatment for sepsis. Cellular therapies using mesenchymal stem cells (MSCs) have been initiated in clinical trials for both ARDS and sepsis based on a wealth of pre-clinical data. However, there remains concern that MSCs may pose a tumor risk when administered to patients. Recent pre-clinical studies have demonstrated the beneficial effects of MSC-derived extracellular vesicles (EVs) for the treatment of acute lung injury (ALI) and sepsis.

**Methods:**

After recovery of initial surgical preparation, pneumonia/sepsis was induced in 14 adult female sheep by the instillation of *Pseudomonas aeruginosa* (~1.0×10^11^ CFU) into the lungs by bronchoscope under anesthesia and analgesia. After the injury, sheep were mechanically ventilated and continuously monitored for 24 h in a conscious state in an ICU setting. After the injury, sheep were randomly allocated into two groups: Control, septic sheep treated with vehicle, n=7; and Treatment, septic sheep treated with MSC-EVs, n=7. MSC-EVs infusions (4ml) were given intravenously one hour after the injury.

**Results:**

The infusion of MSCs-EVs was well tolerated without adverse events. PaO_2_/FiO_2_ ratio in the treatment group tended to be higher than the control from 6 to 21 h after the lung injury, with no significant differences between the groups. No significant differences were found between the two groups in other pulmonary functions. Although vasopressor requirement in the treatment group tended to be lower than in the control, the net fluid balance was similarly increased in both groups as the severity of sepsis progressed. The variables reflecting microvascular hyperpermeability were comparable in both groups.

**Conclusion:**

We have previously demonstrated the beneficial effects of bone marrow-derived MSCs (10×10^6^ cells/kg) in the same model of sepsis. However, despite some improvement in pulmonary gas exchange, the present study demonstrated that EVs isolated from the same amount of bone marrow-derived MSCs failed to attenuate the severity of multiorgan dysfunctions.

## 1 Introduction

The pathogenesis of sepsis involves an imbalance between the pro-inflammatory and anti-inflammatory components of the immune system. It leads to an overproduction of pro-inflammatory cytokines and an overactivation of the immune system (1), a compromised anti-inflammatory state leading to immunosuppression and hemodynamic and coagulation changes, with cell injury, leading to the development of multiple organ failure (MOD) (2–5). The lung is among the most frequently injured organ in the development of sepsis. As lung injury gets worse clinically, patients will develop acute respiratory distress syndrome (ARDS), with a mortality rate of up to 40% (6, 7). Moreover, the mortality rate for sepsis in the intensive care unit is 40 to 60% (8–11). Currently, there are no effective treatments for sepsis. Because the pathogenesis of sepsis is extremely complex, the ideal therapy for sepsis would need to combine multiple targets, including early immunomodulation, cell protection, and prevention of end-organ damage. Based on extensive pre-clinical data, cell-based therapy with mesenchymal stem cells (MSC) has entered clinical trials for both ARDS and sepsis (12, 13). However, there remain long-term concerns about tumor risk in patients with administration of up to 10 million MSC/kg of body weight per treatment. Recent studies demonstrating the efficacy of MSC-EVs in pre-clinical studies in acute lung injury (ALI) and sepsis suggest a superior therapeutic than MSCs. Although less potent, MSC-EVs have a similar phenotype to their parent cells in suppressing inflammation and increasing bacterial clearance (14–20). Based on their small size (<200 nm), MSC-EVs cause fewer hemodynamic changes with administration than MSCs (with sizes up to 10μm), such as a rise in pulmonary artery pressure. In addition, MSC-EVs do not require a bone marrow transplant facility for storage or a preservative such as DMSO, which may affect the potency of the therapeutic. And most importantly, due to the anuclear properties of the EVs, MSC-EVs pose minimal long-term tumor risk (21, 22). However, many of these pre-clinical studies were performed in rodent models and had limited relevance to human sepsis. Hence, large animal models are required to evaluate respiratory and circulatory dynamics in similar clinical situations. Therefore, we investigated the effect of MSC-EVs in a clinically relevant ovine model of sepsis.

## 2 Materials and methods

### 2.1 Characterization of mesenchymal stem cell

#### 2.1.1 Isolation of mesenchymal stem cell extracellular vesicles

Human bone marrow-derived MSCs were purchased from a National Institutes of Health repository from Texas A&M Health Science Center (Temple, TX) (23).

MSC-EVs were isolated from the conditioned medium of human bone marrow-derived MSCs using ultracentrifugation as we previously described (24, 25). Briefly, MSCs were grown in a T175 flask until 90% confluent and then serum starved in α-MEM supplemented with 0.5% Bovine Albumin Fraction (MP BioMedicals, LLC, Santa Ana, CA, http://www.mpbio.com). After 48 hours, the conditioned medium was collected and centrifuged at 3500 rpm 4℃ for 30 min to remove whole cells, cellular debris, and larger particles and then at 100,000 × g (Beckman Coulter Optima L-100XP Ultracentrifuge) to isolate the MSC-EVs at 4°C for 1 hour. The pellet was resuspended and washed in phosphate-buffered saline (PBS) and then ultracentrifuged again under the same conditions. After the second ultracentrifugation, they were collected at the bottom of tubes, resuspended with PBS (10μl per MSC-EVs released by 1×10^6^ cells), and stored at −80℃.

#### 2.1.2 Characterization and the dose of mesenchymal stem cell extracellular vesicles

In our prior publications, MSC-EVs were well characterized by morphology, size, protein, RNA content, and surface receptors (17). For consistency of preparation, we measured vesicle concentration from different MSC-EV isolations. By Nanosight, different MSC-EV preparations gave vesicle concentration of 10^11^ particles per ml. We chose the dose (4000 ul of MSC-EVs) for the sheep model based on an extrapolation of the dose of MSCs from our previous ex vivo perfused human model to the sheep. Ex vivo perfused human ALI model required a dose of 5-10 million MSCs (26). Also the ex vivo perfused human ALI model required a dose of 100- 200 ul of MSC-EVs for an equivalent effect (27). We have also reported that the sheep model of ALI required a dose of 5-10 million cells/kg (200-400 million MSCs) (28).

#### 2.1.3 Animal model

Fourteen adult female Merino sheep were studied. The care and use of sheep followed the guidelines for using laboratory animals from the National Institutes of Health and the American Physiological Society (29). Approximately three-year-old females weighing 35 to 40 kg were purchased from Talley Ranch, Bastrop, TX. The Institutional Animal Care and Use Committee at the University of Texas Medical Branch approved the protocol for the study. All sheep were screened by a veterinarian and group housed at the Animal Research Center with free access to food and water until the day before the surgical procedures.

#### 2.1.4 Surgical procedures

After at least 14 days of quarantine, sheep were housed in individual cages and transferred to the Translational Intensive Care Unit for the surgical procedures as described previously (30). Briefly, sheep were sedated with intramuscular ketamine (500mg) injection followed by its intravenous bolus injection (300mg; KetaVed; Vedco, St. Joseph, MO). Then, the endotracheal tube was placed with isoflurane inhalation *via* a mask (2–5%; Piramal Healthcare, Digwal, India). Afterward, the anesthesia was maintained with inhaled isoflurane *via* the endotracheal tube to effect (2-5%). For pain control, a subcutaneous injection of long-acting (72 h) buprenorphine (0.1mg/kg; Buprenorphine SR; ZooPharm, Laramie, WY) was given. After shaving the fur and weighing, the sheep were transferred to the operating room. A polyvinyl chloride catheter (Park-Davis, Sandy, UT) was implanted through the right femoral artery to monitor heart rate (HR) and mean arterial pressure (MAP) and draw blood samples. A 7 Fr Swan-Ganz thermodilution catheter (Edwards Lifesciences, Irvine, CA) was inserted into the common pulmonary artery through the right external jugular vein to monitor pulmonary arterial pressure (PAP), pulmonary capillary wedge pressure (PCWP), central venous pressure (CVP), intermittent cardiac output, and core body temperature. A Silastic catheter (Dow Corning, Midland, MI) was inserted into the left atrium of the heart through a left thoracotomy at the fifth intercostal space. After the surgical procedure, anesthesia was discontinued, and sheep were extubated when they could maintain adequate spontaneous breathings and transferred to the ICU. Pre- and post-surgical analgesia were provided with intravenous administration of long-acting (72 h) buprenorphine (0.1mg/kg). During the recovery period of about a week, sheep received intravenous fluid resuscitation (lactated Ringer’s solution; 2 mL·kg body wt-1·h-1) and free access to food and water. All implanted catheters were continuously flushed with heparinized saline using a transducer (Truwave PX4X4; Edwards Lifesciences) and pressure infusor (Clear Cuff MX4710; Smiths Medical) to prevent a clot from forming in the lines.

#### 2.1.5 Induction of pneumonia

After surgical recovery, fasted sheep (24 h) were again sedated with an intravenous bolus injection of ketamine (500mg) followed by a subcutaneous injection of buprenorphine (0.1mg/kg) as pre-and post-surgical analgesia. Then, anesthesia was maintained by isoflurane inhalation (2-5%) through a face-fitting mask. The tracheostomy was performed, a 10 mm tube (Shiley; COVIDIEN) was placed in the trachea, and isoflurane inhalation was switched from the face mask to the tracheostomy tube. A Foley catheter was placed in the urinary bladder of female sheep. Then, a total of 30 mL of live *P. aeruginosa* (PA,~1.0×10^11^ CFU) mixed with saline was instilled into the lungs (10 mL in the right middle, 10 mL in the right lower, and 10 mL in the left lower lobes) by a bronchoscope (model BF-P40; Olympus, Tokyo, Japan). The number of PA bacteria was determined based on our previous studies (31, 32).

#### 2.1.6 Post-injury care

Immediately after the PA instillation, mechanical ventilation (Avea ventilator system; CareFusion, Yorba Linda, CA) started with pressure-regulated volume-controlled mode. The tidal volume (TV), positive end-expiratory pressure (PEEP), and respiratory rate were 12 mL/kg, 5 cmH_2_O, and 20 breaths/min, respectively. FiO_2_ was initially (for three h) set at 100% and further adjusted to keep PaO_2_ around 100 mmHg. The respiratory rate was first set at 20 breaths/min and further adjusted to control PaCO_2_ between 30-40 mmHg. The cardiopulmonary variables were monitored for 24 h in a conscious state. During the study, sheep had free access to food but not water to calculate the fluid balance accurately. To enhance translational aspects of the study, sheep were treated with an antibiotic (Cefazoline, 2g), titrated norepinephrine to keep MAP close to baseline (10 mmHg below the baseline) but not to exceed it, and fluid resuscitated. For the fluid resuscitation, lactated Ringer’s solution was initiated with an initial rate of 2 mL/kg/h, which was further adjusted every 3 h to keep hematocrit close to baseline ( ± 3%).

#### 2.1.7 Mesenchymal stem cell extracellular vesicle treatment

Sheep were studied in pairs to provide side-by-side assessment and were randomized to treatment with MSC-EVs (treatment: n=7) or saline (control: n=7). The MSC-EVs were stored frozen, and on the study day, the vial with MSC-EVs was thawed gently. The total volume (4mL) of a solution containing MSC-EVs was transferred into a sterile infusion bag containing 100 mL of USP-grade 0.9% NaCl using a 14-gauge needle and a syringe. At 1 h after the injury, MSC-EVs were administered by intravenous infusion through a central vein catheter within 30 minutes. Control sheep received 0.9% NaCl infusion at a matching rate. Physiologic measurements were performed at baseline, 0.5, 1, 5, 10, 15, 20, 30, 40, 50, and 60 min after initiation of MSC-EVs infusion in awake sheep.

#### 2.1.8 Measured variables

Cardiopulmonary hemodynamics, mechanical ventilation readouts, fluid input, urine output, and arterial and mixed venous blood gas analysis were recorded at baseline and every three hours after that. In each graph, 0 h was the baseline, and the baseline measurement was performed immediately before bacterial inoculation. Hemodynamic variables included PAP, PCWP, CVP, systolic blood pressure (SBP), MAP, LAP, and HR. (hemodynamic monitor, IntelliVue MP50; Philips Medizin Systeme Boeblingen, Boeblingen, Germany). Cardiac output (CO) was determined three times by standard methods, and two relative values were used to calculate the mean cardiac index (CI). CI and systemic vascular resistance index (SVRI) were calculated according to the standard formula (33). The mechanical ventilation readouts included FiO_2_, TV, respiratory rate (RR), peak and plateau pressure, and dynamic compliance. The mean airway pressure, PaO_2_/FiO_2_ (P/F) ratio, static compliance, and pulmonary shunt fraction (Qs/Qt) were calculated according to the standard formula. The urine output in female sheep was measured *via* a Foley catheter. The blood gas analysis was performed using a blood gas analyzer (RAPID Point 500; Siemens Healthcare, Erlangen, Germany). Blood samples were taken from a femoral artery to determine the white blood cells count (WBC) and C-reactive protein (CRP) and centrifuged under 4,000 rpm at 4℃ for plasma and serum separation to measure creatinine, and total bilirubin. Postmortem, bloodless lung wet-to-dry weight (W/D) ratio was determined by the method described by Pearce et al. (34). Microvascular hyperpermeability was indirectly evaluated by measuring lung extravascular water content and fluid balance.

#### 2.1.9 Euthanasia criteria

The sheep were euthanized at the end of 24 h monitoring or at the time of reaching the euthanasia criteria by using intravenous infusion of ketamine (40mg/kg), xylazine (3.0mg/kg), and buprenorphine (0.01 mg/kg) (35). The euthanasia criteria included reduced mean arterial pressure (MAP) <40 mmHg, reduced heart rate (HR) <40 beats/min, increased PaCO_2_ > 90 mmHg, or decreased PaO_2_ <50 mmHg at 100% FiO_2_ for at least an hour.

#### 2.1.10 Bacterial clearance assay

As previously described (36), 1g of the lung tissue was taken from the dorsal edge of the right middle lobe, and 1g of the spleen was taken during the necropsy and homogenized in 3 mL of 1×PBS. Then, a ten-fold serial dilution was performed on each of the tissue homogenates. 10µL of each tissue homogenate dilution was pipetted and streaked onto Tryptic soy agar (TSA) plates. Bronchial alveolar lavage fluid was also taken from the left lower lobe during the necropsy and pipetted with ten-fold serial dilution onto the TSA plates. The plates were incubated for 24 h at 37°C for bacterial CFU counts (32).

#### 2.1.11 Statistical analysis

All statistical analyses were performed using GraphPad Prism version 9.4.1 (Graph-Pad Software, Inc., La Jolla, CA). Results were compared between the groups at each time point by a two-way ANOVA with a mixed-effects model with *post hoc* Bonferroni or Sidak multiple comparison tests. The values measured at a single time point were compared by unpaired t-test or Mann–Whitney U test, based on the normality of the data distribution (Shapiro–Wilk test). All values are expressed as mean ± standard error of the mean (mean ± SEM). Statistical significance was considered for p-value < 0.05.

## 3 Results

### 3.1 Mortality rate

**Figure 1:**
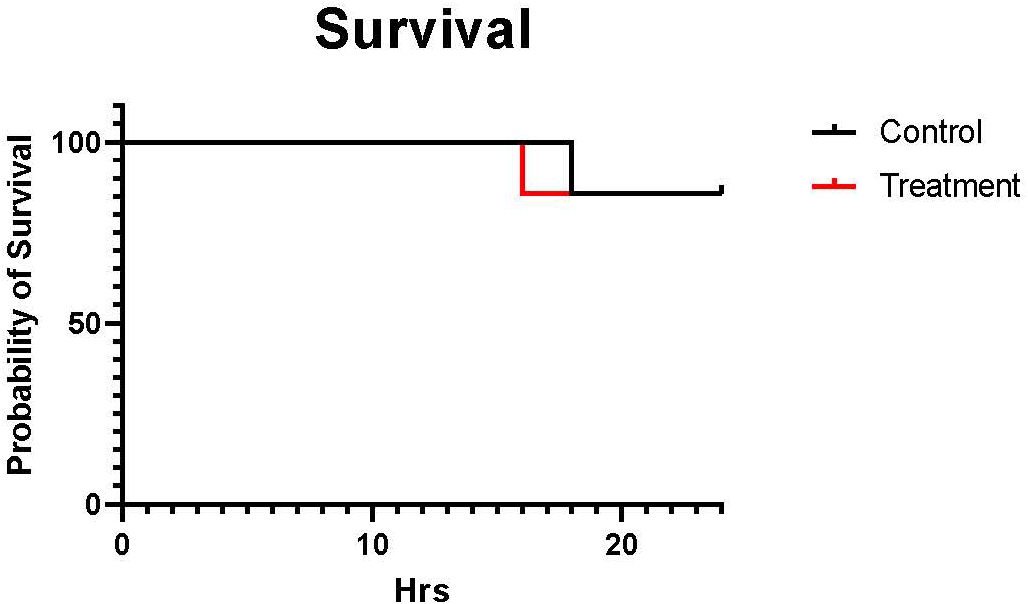
The Kaplan-Meier curve of the mortality rate. The black line shows control (n=7 at 0-18 h and 6 at 19-24 h), and the red line shows treatment (n=7 at 0-16 h and 6 at 17-24 h). The mortality rate was calculated by dividing the number of sheep that not survived within 24 h by the total number of sheep in each group. The mortality rate was similar between males and females (14% vs. 14%).

The mortality rate was calculated by dividing the number of non-survival (euthanized upon reaching euthanasia criteria) sheep within 24 h by the total number of sheep in each group. Six sheep out of seven survived in both groups during the study period. One control sheep were euthanized at 18 h, and one treatment sheep was euthanized at 16 h upon reaching the euthanasia criteria. The mortality rate was similar between control and treatment (14% vs. 14%) (**Figure 1**).

### 3.2 The severity of pulmonary dysfunction

**Figure 2:**
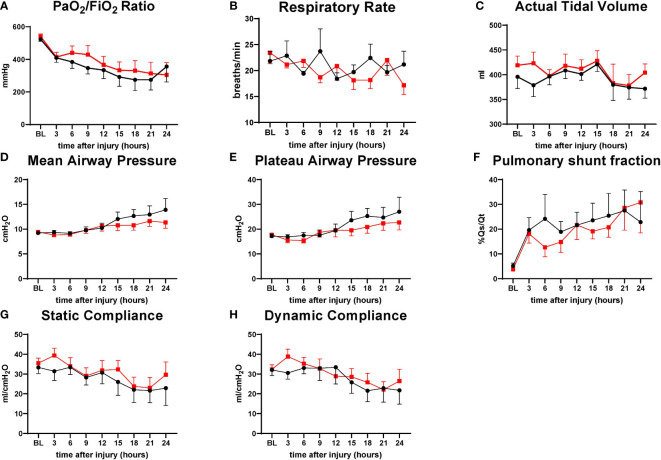
The severity of lung injury. ● Control (n=7 at 0-18 h and 6 at 19-24 h), ■ Treatment (n=7 at 0-16 h and 6 at 17-24 h. There were no differences in pulmonary function. PaO_2_/FiO_2_ ratio (P/F ratio) **(A)**, respiratory rate (RR) **(B)**, actual tidal volume **(C)**, mean airway pressure **(D)**, plateau airway pressure **(E)**, pulmonary shunt fraction (Qs/Qt) **(F)**, static compliance **(G)**, dynamic compliance **(H)**. Data are expressed as mean ± SEM.

No significant differences between the groups were found in the variables evaluating pulmonary functions. There was a tendency for the treatment group to have a higher PaO_2_/FiO_2_ ratio and static compliance from 6 h to 21 h throughout the study period. Mean airway pressure and plateau airway pressure tended to be higher in the control group sheep from 15 h to 24 h. Both groups were comparable in respiratory rate, actual tidal volume, pulmonary shunt fraction, and dynamic compliance (**Figure 2**).

### 3.3 Hemodynamic changes

**Figure 3:**
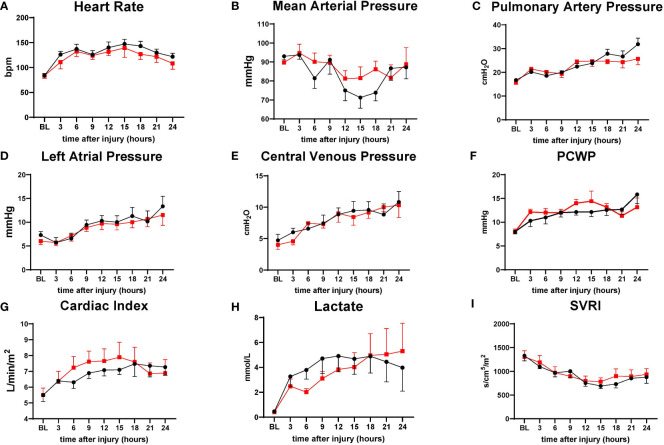
The severity of lung injury. ● Control (n=7 at 0-18 h and 6 at 19-24 h), ■ Treatment (n=7 at 0-16 h and 6 at 17-24 h). There were no differences in hemodynamic changes. Heart rate (HR) **(A)**, mean arterial pressure (MAP) **(B)**, pulmonary artery pressure (PAP) **(C)**, left atrial pressure (LAP) **(D)**, central venous pressure (CVP) **(E)**, pulmonary capillary wedge pressure (PCWP) **(F)**, cardiac index (CI) **(G)**, lactate **(H)**, systemic vascular resistance index (SVRI) **(I)**. Data are expressed as mean ± SEM.

Hemodynamic variables such as HR PAP, LAP, CVP, PWCP, lactate, and SVRI are comparable in both groups. The treatment group tended to have a slightly higher MAP from 12 h to 18 h and Cardiac Index from 6 h to 18 h than the control; however, no significant differences were found between the variables (**Figure 3**).

### 3.4 Microvascular hyperpermeability

**Figure 4:**
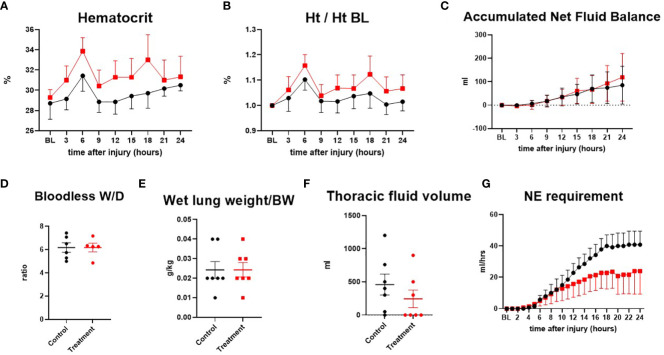
The severity of lung injury. ● Control (n=7 at 0-18 h and 6 at 19-24 h), ■ Treatment (n=7 at 0-16 h and 6 at 17-24 h). Hematocrit (Ht) **(A)**, hematocrit/hematocrit at baseline (Ht/Ht BL) **(B)**, accumulated net fluid balance **(C)**, bloodless wet-to-dry weight ratio (W/D ratio) **(D)**, wet lung weight/body weight **(E)**, thoracic fluid volume **(F)**, Norepinephrine requirement **(G)**. Data are expressed as mean ± SEM.

The hematocrit was shown in actual numbers and numbers adjusted to the baseline as a percentage. Although treatment sheep tended to have higher hematocrit (one sheep in control group had an unusual pattern of low hematocrit, resulting in lower mean hematocrit value) at baseline and post-injury time points, overall changes in hematocrit were comparable in both groups. The net fluid balance, a measure of accumulated fluid over time, was similarly increased in both groups as the severity of sepsis progressed. The lung bloodless wet-to-dry weight ratio, wet lung weight per body weight, and thoracic fluid volume were comparable in both groups, and no significant difference was found between the groups. Accumulation of exudate in the thoracic cavity tended to be lower in the treated group. Control sheep required more vasopressor to maintain MAP than treatment sheep after 10 h post-injury (**Figure 4**).

### 3.5 Altered mental status

**Figure 5:**
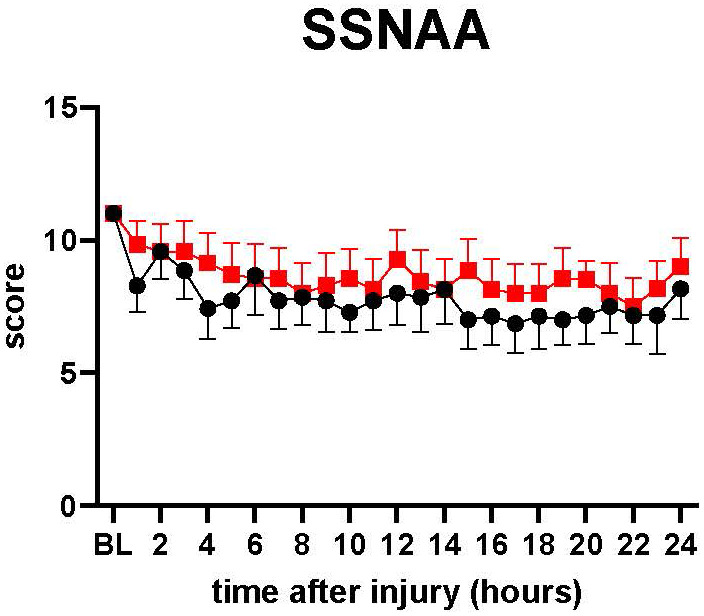
SSNAA: A simplified sheep neurological/alertness assessment scale. The severity of lung injury. ● Control (n=7 at 0-18 h and 6 at 19-24 h), ■ Treatment (n=7 at 0-16 h and 6 at 17-24 h). The SSNAA score tended to be slightly higher in treatment than in control from 15 h to 24 h, but there was no significant difference between the groups. Data are expressed as mean ± SEM.

The neurological status of the animal was assessed by the Simplified Sheep Neurological/Alertness Assessment score (SSNAA). The SSNAA is scored by summing the scores for response to approach (1,2,4), response to sound (1,2,4), and response to mechanical stimulations (0,1,2,3) (total score 2-11) (**Supplement Table 1**). The SSNAA score tended to be slightly higher in treatment than in control from 15 h to 24 h (**Figure 5**).

### 3.6 Bacterial clearance assay

**Figure 6:**
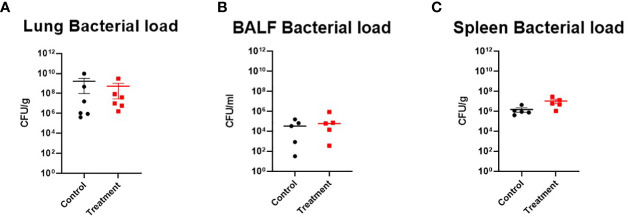
Bacterial clearance assay. **(A)** Lung bacterial load ● Control (n=6), ■ Treatment (n=6), **(B)** BALF bacterial load ● Control (n=5), ■ Treatment (n=5), **(C)** Spleen bacterial load ● Control (n=5), ■ Treatment (n=5). There was no significant difference between the groups in the bacterial clearance assay. Data are expressed as mean ± SEM.

The number of bacteria in lung tissue at 24 h after PA instillation in control was 1.68×10^9^ ± 1.58×10^9^ CFUs/g, and in the treatment group, it was 5.48×10^8^± 5.19×10^8^ CFUs/g. The number of bacteria in the spleen in control was 1.52×10^6^ ± 7.23×10^5^ CFUs/g, and in treatment, it was 1.08×10^7^ ± 4.95×10^6^ CFUs/g. The number of bacteria in BALF in control was 5.13×10^4^ ± 2.90×10^4^ CFUs/g, and in treatment, it was 2.03×10^5^ ± 1.66×10^5^ CFUs/g. Although treatment sheep tended to have lower bacterial numbers in lung tissue than control sheep, treatment sheep tended to have higher bacteria numbers in the spleen and BALF, and no significant difference was found between the groups in each tissue (**Figure 6**).

### 3.7 Inflammatory marker

**Figure 7:**
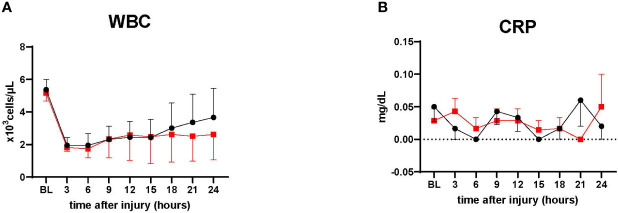
Inflammatory marker. **(A)** White blood cells count (WBC), ● Control (n=4 at 0-24 h), ■ Treatment (n=4 at 0-24 h). **(B)** C-reactive protein measurement. ● Control (n=7 at 0-18 h and 6 at 19-24 h), ■ Treatment (n=7 at 0-16 h and 6 at 17-24 h). WBC in the treatment tended to be lower than the control after 18 h. CRP showed relatively low in both groups, with no significant differences. Data are expressed as mean ± SEM.

WBC in the treatment tended to be lower than the control after 18 h (n=4, each group). However, no statistical significance was noted between the groups. CRP changes were comparable in both groups (**Figure 7**)

## 4 Discussion

Cell-based therapy with stem cells may be important for physiologic maintenance and organ repair in the event of injury. This has been extensively studied for the lung. Stem cell therapy using mesenchymal stem cells (MSCs), endothelial progenitor cells (EPCs), embryonic stem cells (ESCs), and induced pluripotent stem cells (iPSCs) are novel treatment options for ALI. MSCs possess multipotency and repair functions and can be harvested from almost every postnatal tissue. Their easier accessibility, improved safety profile, and nonexistent ethical concerns make them a superior candidate for the cell-based therapy compared to ESCs and iPSCs (37). Special attractiveness arises from the immune-privileged status of MSCs. They do not trigger a host response or cell rejection because they are less sensitive to the effects of HLA-II expression by inflammatory IFN-γ (38). In various pre-clinical ALI models, MSCs have been shown to secrete multiple paracrine factors that reduce lung endothelial and epithelial permeability, decrease inflammation, promote tissue repair, inhibit bacterial growth, and ultimately reduce mortality. However, concerns about using stem cells, specifically the risk of iatrogenic tumor formation, remain unresolved. Currently, the accumulating evidence suggests that new cell-free therapies involving MSC-derived conditioning medium and extracellular vesicles released from MSCs might be alternative therapies.

In comparison with MSCs, MSC-EVs possess hypoimmunogenic properties, low tumorigenesis, and higher stability (39). Functions similar to those of their parental cells, such as antimicrobial effects, immunomodulatory properties, and the ability to repair damaged tissues, have been observed in MSC-EVs (40).The paracrine effect mediated by secreted growth factors, cytokines, and extracellular vesicles is mainly responsible for the efficacy of MSCs (41). MSC-EVs have been identified as the main parts responsible for the paracrine effect. They transfer functional molecules such as messenger RNA (mRNA), microRNA (miRNA), lipids, mitochondria, and proteins to tissue-specific cells in need of repair. These molecules in MSC-EVs play a critical role in modulating immune responses and repairing lung injury in ALI/ARDS. MiRNAs in MSC-EVs have been considered critical to exert efficacy in sepsis (14).

Some researchers showed that intratracheal administration of MSC-EVs had therapeutic effects in hyperoxia-induced lung injury, demonstrating that MSC-EVs could ameliorate impaired alveolarization in both short and long-term bronchopulmonary dysplasia models and activate M2 macrophages (42, 43). The effects of MSC-EVs on COVID-19, a pandemic disease for which no specific antiviral medication, is being investigated in two clinical trials. MSC-EVs are administered intravenously (NCT04798716) or by inhalation (NCT04276987). Allogeneic bone marrow MSC-derived exosomes (ExoFloTM) were shown to be safe and effective in restoring oxygenation, downregulating cytokine storm, and reconstituting immunity in severe COVID-19 patients in a prospective, non-randomized, open-label cohort study (44).

The results of our present study demonstrated that the use of MSCs-EVs failed to attenuate pneumonia/sepsis-induced multiorgan dysfunctions in a clinically relevant ovine model despite some tendency in improving pulmonary gas exchange and reducing the vasopressor requirement to maintain the blood pressure. Currently, the reason for the inefficiency of these EVs is not clear. As mentioned, we have treated septic sheep with EVs harvested from 10×10^6^/kg body weight one time, 1 h after the injury. It is possible that the dose of the EVs was too small, or repeated treatment was needed. It is also possible that IV-administered MSCs continuously secrete EVs while circulating within the body, thus producing more EVs than ones harvested from 10^6^/kg cells at a given time. This notion is supported by findings by Silva et al., who reported that EVs were less effective than parental MSCs at reducing lung injury (45). Monsel et al. reported that higher EVs concentration was needed to obtain similar therapeutic effects as MSCs (46). Zhu and collaborators reported a modest effect of EVs when they were given based on the final MSC cell count. The authors achieved enhanced therapeutic effect only with the increased doses of EVs (16). In general, we estimate that MSC-EVs are 5-10× less potent than MSCs in pre-clinical ALI/sepsis models.

The homing of the EVs to the site injury can also be of concern. The limitation of the study is also related to the lack of information on the kinetics of the IV-injected EVs within the body, especially in the septic environment. Nevertheless, we report that EVs harvested from 10^6^/kg cells do not produce benefits against PA-induced sepsis. Future dose-dependent studies should be carried out to eliminate the limitations mentioned above. Another limitation of this study is the duration of our studies was relatively short (24 h), which precluded comparison of the extent of recovery over time in both groups and the more prolonged efficacy of MSC-EVs in the septic sheep.

Despite the equivocal results, there is still the potential for using MSC-EVs clinically for ARDS or sepsis. For example, Sengupta et al. (44) found improved oxygenation with an average pressure of arterial oxygen to fraction of inspired oxygen ratio (PaO_2_/FiO_2_) increase of 192% (P < 0.001) in 24 patients with COVID-associated ARDS who were administered 15 mL of ExoFlo (Direct Biologics, LLC). Their study was a multicenter, double-blinded, placebo-controlled, randomized control trial where patients received normal saline 90 mL or ExoFlo 10-15 mL, which contains approximately 800–1,200 billion EVs released by MSCs (44). These studies raise the question of whether MSC-EVs may be better suited as a potential therapeutic for patients with sterile ARDS induced by various etiology factors, i.e., ventilator-induced lung injury, trauma, transfusion-associated ALI, etc. Regardless, the issue of potency and pharmacokinetics of administered MSC-EVs remains the main barrier to bringing this very promising therapeutic to clinical use.

## 5 Conclusion

We have previously demonstrated the beneficial effects of bone marrow-derived MSCs (10×10^6^ cells/kg) in the same model of sepsis as well as the smoke inhalation-induced ARDS model. The results of our present study indicate that EVs harvested from the same amount of MSCs do not produce equivalent efficacy, suggesting that higher doses of EVs are required to be isolated from a greater number of MSCs to achieve the same therapeutic effects obtained by parent MSCs that exerted benefits.

## Data availability statement

The raw data supporting the conclusions of this article will be made available by the authors, without undue reservation.

## Ethics statement

The animal study was reviewed and approved by The Institutional Animal Care and Use Committee at the University of Texas Medical Branch.

## Author contributions

KeH and PE conceived the experiment. KaH, NB, and KeH conducted the experiment and analyzed the data. MS, QH, and JL contributed to the preparation of MSC-EVs. KeH and MS drafted the manuscript and figures and carried out the literature search. JL and PE carried out the manuscript modification. DP helped perform the manuscript with constructive discussions. TH helped with bacterial clearance assay and manuscript editing. All authors contributed to the article and approved the submitted version.

## References

[1] Prescott HC Angus DC. Enhancing recovery from sepsis. JAMA. (2018) 319(1):62. doi: 10.1001/jama.2017.17687 29297082 PMC5839473

[2] Hotchkiss RS Karl IE. The pathophysiology and treatment of sepsis. New Engl J Med (2003) 348(2):138–50. doi: 10.1056/NEJMra021333 12519925

[3] Oberholzer A Oberholzer C Moldawer LL. Sepsis syndromes: understanding the role of innate and acquired immunity. Shock. (2001) 16(2):83–96. doi: 10.1097/00024382-200116020-00001 11508871

[4] Gyawali B Ramakrishna K Dhamoon AS. Sepsis: the evolution in definition, pathophysiology, and management. SAGE Open Med (2019) 7:205031211983504. doi: 10.1177/2050312119835043 PMC6429642 30915218

[5] Caraballo C Jaimes F. Organ dysfunction in sepsis: an ominous trajectory from infection to death. Yale J Biol Med (2019) 92(4):629–40. PMC6913810 31866778

[6] Lee JH Park J Lee J-W. Therapeutic use of mesenchymal stem cell-derived extracellular vesicles in acute lung injury. Transfusion. (2019) 59(S1):876–83. doi: 10.1111/trf.14838 PMC6368889 30383895

[7] Englert JA Bobba C Baron RM. Integrating molecular pathogenesis and clinical translation in sepsis-induced acute respiratory distress syndrome. JCI Insight (2019) 4(2):e124061. doi: 10.1172/jci.insight.124061 30674720 PMC6413834

[8] Augustine S Avey MT Harrison B Locke T Ghannad M Moher D. Mesenchymal stromal cell therapy in bronchopulmonary dysplasia: systematic review and meta-analysis of pre-clinical studies. Stem Cells Trans Med (2017) 6(12):2079–93. doi: 10.1002/sctm.17-0126 PMC5702524 29045045

[9] Seymour CW Liu VX Iwashyna TJ Brunkhorst FM Rea TD Scherag A. Assessment of clinical criteria for sepsis. JAMA. (2016) 315(8):762. doi: 10.1001/jama.2016.0288 26903335 PMC5433435

[10] Singer M Deutschman CS Seymour CW Shankar-Hari M Annane D Bauer M. The third international consensus definitions for sepsis and septic shock (Sepsis-3). JAMA. (2016) 315(8):801. doi: 10.1001/jama.2016.0287 26903338 PMC4968574

[11] Brun-Buisson C Roudot-Thoraval FO Girou E Grenier-Sennelier C Durand-Zaleski I. The costs of septic syndromes in the intensive care unit and influence of hospital-acquired sepsis. Intensive Care Med (2003) 29(9):1464–71. doi: 10.1007/s00134-003-1877-x 12856120

[12] Monsel A Zhu Y-G Gennai S Hao Q Liu J Lee JW. Cell-based therapy for acute organ injury. Anesthesiology. (2014) 121(5):1099–121. doi: 10.1097/ALN.0000000000000446 PMC4206665 25211170

[13] Matthay MA Calfee CS Zhuo H Thompson BT Wilson JG Levitt JE. Treatment with allogeneic mesenchymal stromal cells for moderate to severe acute respiratory distress syndrome (START study): a randomised phase 2a safety trial. Lancet Respir Med (2019) 7(2):154–62. doi: 10.1016/S2213-2600(18)30418-1 PMC7597675 30455077

[14] You J Fu Z Zou L. Mechanism and potential of extracellular vesicles derived from mesenchymal stem cells for the treatment of infectious diseases. Front Microbiol (2021) 12:761338. doi: 10.3389/fmicb.2021.761338 34764947 PMC8576143

[15] Hu S Park J Liu A Lee J Zhang X Hao Q. Mesenchymal stem cell microvesicles restore protein permeability across primary cultures of injured human lung microvascular endothelial cells. Stem Cells Trans Med (2018) 7(8):615–24. doi: 10.1002/sctm.17-0278 PMC6090509 29737632

[16] Zhu YG Feng XM Abbott J Fang XH Hao Q Monsel A. Human mesenchymal stem cell microvesicles for treatment of escherichia coli endotoxin-induced acute lung injury in mice. Stem Cells (2014) 32(1):116–25. doi: 10.1002/stem.1504 PMC3947321 23939814

[17] Hao Q Gudapati V Monsel A Park JH Hu S Kato H. Mesenchymal stem cell-derived extracellular vesicles decrease lung injury in mice. J Immunol (2019) 203(7):1961–72. doi: 10.4049/jimmunol.1801534 PMC6760999 31451675

[18] Song Y Dou H Li X Zhao X Li Y Liu D. Exosomal miR-146a contributes to the enhanced therapeutic efficacy of interleukin-1β-Primed mesenchymal stem cells against sepsis. Stem Cells (2017) 35(5):1208–21. doi: 10.1002/stem.2564 28090688

[19] Yao M Cui B Zhang W Ma W Zhao G Xing L. Exosomal miR-21 secreted by IL-1β-primed-mesenchymal stem cells induces macrophage M2 polarization and ameliorates sepsis. Life Sci (2021) 264:118658. doi: 10.1016/j.lfs.2020.118658 33115604

[20] Wang X Gu H Qin D Yang L Huang W Essandoh K. Exosomal miR-223 contributes to mesenchymal stem cell-elicited cardioprotection in polymicrobial sepsis. Sci Rep (2015) 5(1):13721. doi: 10.1038/srep13721 26348153 PMC4562230

[21] Hwang W Shimizu M Lee JW. Role of extracellular vesicles in severe pneumonia and sepsis. Expert Opin Biol Ther (2022) 22(6):747–62. doi: 10.1080/14712598.2022.2066470 PMC9971738 35418256

[22] Liu A Zhang X He H Zhou L Naito Y Sugita S. Therapeutic potential of mesenchymal stem/stromal cell-derived secretome and vesicles for lung injury and disease. Expert Opin Biol Ther (2020) 20(2):125–40. doi: 10.1080/14712598.2020.1689954 PMC6981051 31701782

[23] Dominici M Le Blanc K Mueller I Slaper-Cortenbach I Marini F Krause D. Minimal criteria for defining multipotent mesenchymal stromal cells. the international society for cellular therapy position statement. Cytotherapy. (2006) 8(4):315–7. doi: 10.1080/14653240600855905 16923606

[24] Monsel A Zhu Y-G Gennai S Hao Q Hu S Rouby J-J. Therapeutic effects of human mesenchymal stem cell–derived microvesicles in severe pneumonia in mice. Am J Respir Crit Care Med (2015) 192(3):324–36. doi: 10.1164/rccm.201410-1765OC PMC4584251 26067592

[25] Bruno S Grange C Collino F Deregibus MC Cantaluppi V Biancone L. Microvesicles derived from mesenchymal stem cells enhance survival in a lethal model of acute kidney injury. PloS One (2012) 7(3):e33115. doi: 10.1371/journal.pone.0033115 22431999 PMC3303802

[26] Lee JW Krasnodembskaya A Mckenna DH Song Y Abbott J Matthay MA. Therapeutic effects of human mesenchymal stem cells in ex vivo human lungs injured with live bacteria. Am J Respir Crit Care Med (2013) 187(7):751–60. doi: 10.1164/rccm.201206-0990OC PMC3678109 23292883

[27] Park J Kim S Lim H Liu A Hu S Lee J. Therapeutic effects of human mesenchymal stem cell microvesicles in an ex vivo perfused human lung injured with severe e. coli pneumonia. Thorax. (2019) 74(1):43–50. doi: 10.1136/thoraxjnl-2018-211576 30076187 PMC6295323

[28] Asmussen S Ito H Traber DL Lee JW Cox RA Hawkins HK. Human mesenchymal stem cells reduce the severity of acute lung injury in a sheep model of bacterial pneumonia. Thorax. (2014) 69(9):819–25. doi: 10.1136/thoraxjnl-2013-204980 PMC4284068 24891325

[29] Bayne K. Revised guide for the care and use of laboratory animals available. American physiological society. Physiologist (1996) 39(4):199, 208–199, 211. 8854724

[30] Yaghouby F Daluwatte C Fukuda S Nelson C Salsbury J Kinsky M. Progression and variability of physiologic deterioration in an ovine model of lung infection sepsis. J Appl Physiol (1985) (2017) 123(1):172–81. doi: 10.1152/japplphysiol.00122.2017 28473609

[31] Sousse LE Jonkam CC Traber DL Hawkins HK Rehberg SW Traber LD. Pseudomonas aeruginosa is associated with increased lung cytokines and asymmetric dimethylarginine compared with methicillin-resistant staphylococcus aureus. Shock. (2011) 36(5):466–70. doi: 10.1097/SHK.0b013e3182336b45 PMC3742026 21921834

[32] Fukuda S Ihara K Bohannon JK Hernandez A Patil NK Luan L. Monophosphoryl lipid a attenuates multiorgan dysfunction during post-burn pseudomonas aeruginosa pneumonia in sheep. Shock. (2020) 53(3):307–16. doi: 10.1097/SHK.0000000000001364 PMC6937402 31045990

[33] Enkhbaatar P Joncam C Traber L Nakano Y Wang J Lange M. Novel ovine model of methicillin-resistant staphylococcus aureus-induced pneumonia and sepsis. Shock. (2008) 29(5):642–9. doi: 10.1097/SHK.0b013e318158125b 17885644

[34] Pearce ML Yamashita J Beazell J. Measurement of pulmonary edema. Circ Res (1965) 16:482–8. doi: 10.1161/01.RES.16.5.482 14289157

[35] Fukuda S Niimi Y Hirasawa Y Manyeza ER Garner CE Southan G. Modulation of oxidative and nitrosative stress attenuates microvascular hyperpermeability in ovine model of pseudomonas aeruginosa sepsis. Sci Rep (2021) 11(1):23966. doi: 10.1038/s41598-021-03320-w 34907252 PMC8671546

[36] Fukuda S Ihara K Andersen CR Randolph AC Nelson CL Zeng Y. Modulation of peroxynitrite reduces norepinephrine requirements in ovine MRSA septic shock. Shock. (2019) 52(5):e92–e9. doi: 10.1097/shk.0000000000001297 PMC11676001 30499879

[37] Yang J Jia Z. Cell-based therapy in lung regenerative medicine. Regenerative Med Res (2014) 2(1):7. doi: 10.1186/2050-490x-2-7 PMC4389643 25984335

[38] Gao F Chiu SM Motan DAL Zhang Z Chen L Ji H-L. Mesenchymal stem cells and immunomodulation: current status and future prospects. Cell Death Disease (2016) 7(1):e2062–e. doi: 10.1038/cddis.2015.327 PMC4816164 26794657

[39] Trounson A McDonald C. Stem cell therapies in clinical trials: progress and challenges. Cell Stem Cell (2015) 17(1):11–22. doi: 10.1016/j.stem.2015.06.007 26140604

[40] Fu X Liu G Halim A Ju Y Luo Q Song AG. Mesenchymal stem cell migration and tissue repair. Cells. (2019) 8(8):784. doi: 10.3390/cells8080784 31357692 PMC6721499

[41] Paliwal S Chaudhuri R Agrawal A Mohanty S. Regenerative abilities of mesenchymal stem cells through mitochondrial transfer. J BioMed Sci (2018) 25(1):31. doi: 10.1186/s12929-018-0429-1 29602309 PMC5877369

[42] Porzionato A Zaramella P Dedja A Guidolin D Van Wemmel K Macchi V. Intratracheal administration of clinical-grade mesenchymal stem cell-derived extracellular vesicles reduces lung injury in a rat model of bronchopulmonary dysplasia. Am J Physiology-Lung Cell Mol Physiol (2019) 316(1):L6–L19. doi: 10.1152/ajplung.00109.2018 30284924

[43] Porzionato A Zaramella P Dedja A Guidolin D Bonadies L Macchi V. Intratracheal administration of mesenchymal stem cell-derived extracellular vesicles reduces lung injuries in a chronic rat model of bronchopulmonary dysplasia. Am J Physiol Lung Cell Mol Physiol (2021) 320(5):L688–l704. doi: 10.1152/ajplung.00148.2020 33502939

[44] Sengupta V Sengupta S Lazo A Woods P Nolan A Bremer N. Exosomes derived from bone marrow mesenchymal stem cells as treatment for severe COVID-19. Stem Cells Dev (2020) 29(12):747–54. doi: 10.1089/scd.2020.0080 PMC7310206 32380908

[45] Silva JD de Castro LL Braga CL Oliveira GP Trivelin SA Barbosa-Junior CM. Mesenchymal stromal cells are more effective than their extracellular vesicles at reducing lung injury regardless of acute respiratory distress syndrome etiology. Stem Cells Int (2019) 2019:8262849. doi: 10.1155/2019/8262849 31531026 PMC6720722

[46] Monsel A Zhu Y-G Gudapati V Lim H Lee JW. Mesenchymal stem cell derived secretome and extracellular vesicles for acute lung injury and other inflammatory lung diseases. Expert Opin Biol Ther (2016) 16(7):859–71. doi: 10.1517/14712598.2016.1170804 PMC5280876 27011289

