# Supplementary figures and images for: Mesenchymal stem cell-derived exosomes for treatment of sepsis
Source: Front Immunol. 2023 Apr 26;14:1136964. doi: 10.3389/fimmu.2023.1136964 (PMC10169690; doi:10.3389/fimmu.2023.1136964)

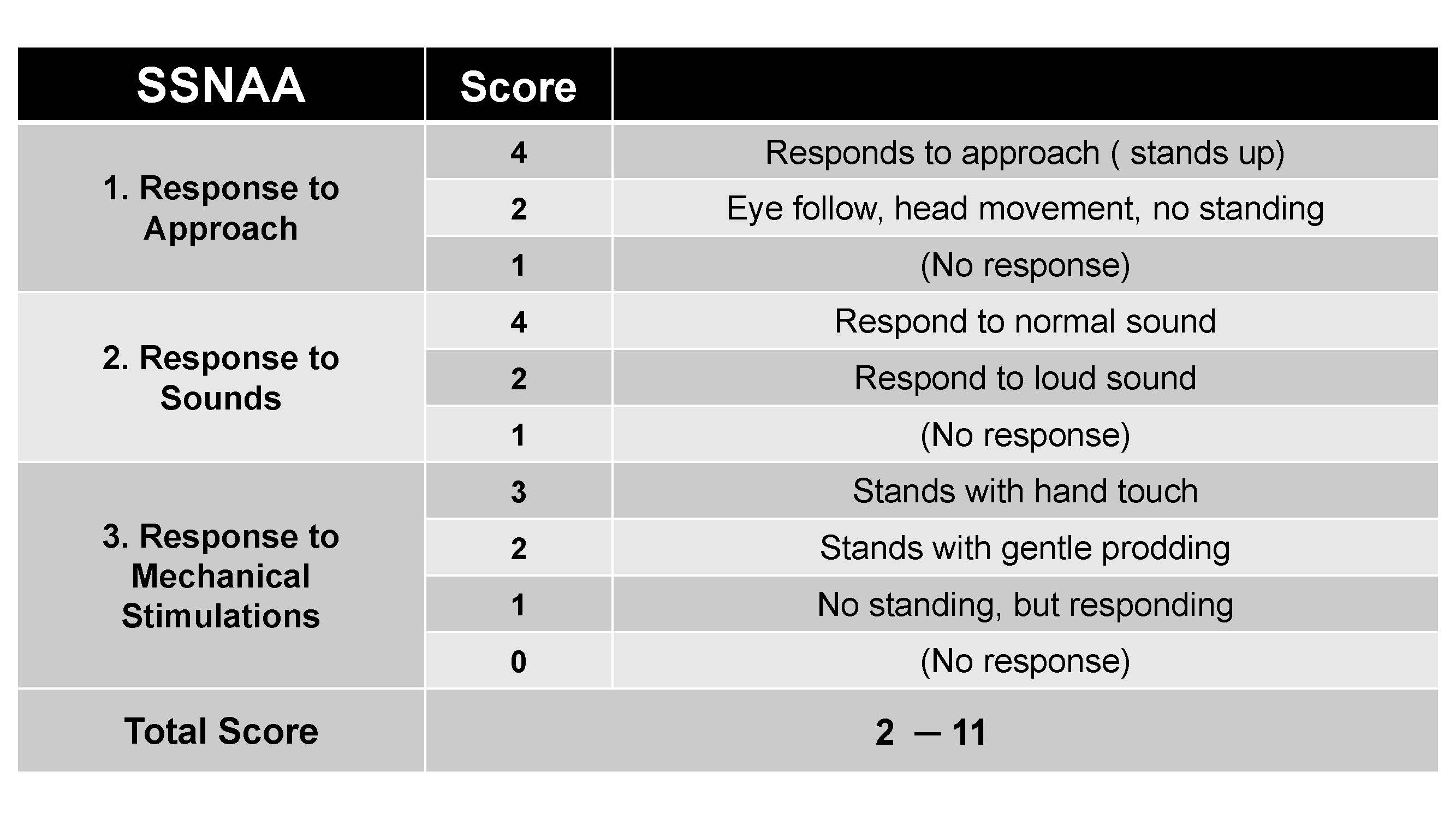

Supplement: Supplementary file 1 [file Image_1.jpeg]
